# Toll-Like Receptors and RIG-I-Like Receptors Play Important Roles in Resisting Flavivirus

**DOI:** 10.1155/2018/6106582

**Published:** 2018-05-14

**Authors:** Hong-Yan Guo, Xing-Cui Zhang, Ren-Yong Jia

**Affiliations:** ^1^Research Center of Avian Disease, College of Veterinary Medicine, Sichuan Agricultural University, Wenjiang District, Chengdu, 611130 Sichuan Province, China; ^2^Institute of Preventive Veterinary Medicine, Sichuan Agricultural University, Wenjiang District, Chengdu, 611130 Sichuan Province, China; ^3^Key Laboratory of Animal Disease and Human Health of Sichuan Province, Wenjiang District, Chengdu, 611130 Sichuan Province, China

## Abstract

Flaviviridae family is a class of single-stranded RNA virus, which is fatal to human and animals and mainly prevalent in subtropic and tropic countries. Even though people and animals are barraged with flavivirus infection every year, we have not invented either vaccines or antiviral for most flavivirus infections yet. Innate immunity is the first line of defense in resisting pathogen invasion, serving an important role in a resisting virus. Toll-like receptors (TLRs) and retinoic acid-inducible gene I- (RIG-I-) like receptors (RLRs) are crucial pattern recognition receptors (PRRs) that play essential roles in recognizing and clearing pathogens, including resisting flavivirus. In the present review, we provide a significant reference for further research on the function of innate immunity in resisting flavivirus.

## 1. Introduction

Flaviviridae family is a class of positive-sense single-stranded RNA virus, in which the virus particle size is 40~60 nm, encoding at least three structural proteins (C, M/prM, and E) and seven nonstructural proteins (NS1, NS2A, NS2B, NS3, NS4A, NS4B, and NS5) [1]. There has been a noncoding region (UTR) in the 5′ end and 3′ end of Flaviviridae, with methylation cap structure in the 5′ end, but no poly(A) sequence present in the 3′ end [2]. Flaviviridae is composed of four genera: *Flavivirus*, *Pestivirus*, *Pegvirus*, and *Hepacivirus* [3], and causes several diseases and mortality in humans and animals. Flavivirus contains over 70 viruses including dengue virus (DENV), West Nile virus (WNV), Zika virus (ZIKV), Japanese encephalitis virus (JEV) [4], and avian Tembusu virus (ATMUV) [5], which are primarily transmitted via arthropods like mosquitoes and ticks.

According to the reports, the NS protein of Flaviviridae serves principal roles in virus replication and the interaction between hosts and pathogens [6, 7]. From these NS proteins, we know the function of NS3 the most. NS3 is a multifunctional enzyme which is associated with the hydrolysis process of capsid proteins and various DNA- and RNA-related biochemical reactions such as translation, transcription, editing, reorganization, and replication [8–10]. Furthermore, the NS3 of hepatitis C virus (HCV) and pestivirus is responsible for hydrolyzing downstream nonstructural proteins [11], which can be enhanced by NS4A [12]. Unlike NS3, the function of other NS proteins of Flaviviridae has more details to explore. The specific function of NS1 has not been illuminated; however, researchers believe it is mainly an immune factor during virus infection and may be involved in the replication of virus RNA and may be related to the cytopathic effect (CPE) [13]. NS2 and NS4 can be further processed into two mature NS (NS2A and NS2B and NS4A and NS4B), in which NS2 may be a major factor that leads to CPE [14]. While NS4A can guide viral RNA synthesis through involvement in the formation of a multiprotein replication complex, NS4 can also stabilize the interaction between NS3 protease and other nonstructural proteins [15] and is critical for NS5A maturation [16]. There are studies confirming that NS5 accompanied with NS3 can initiate the replication of the virus [17]; however, the function of NS5A in viral replication has not been explored.

Innate immunity is the first line of defense in resisting pathogens, which plays an indispensable role against pathogens. Pattern recognition receptor (PRR) is an essential immune receptor which can realize the conservative sequence of viruses and then target innate immunity [18]. So far, two PRRs, toll-like receptors (TLRs) and retinoic acid-inducible gene I- (RIG-I-) like receptors (RLRs) [18, 19], are identified to have a crucial function in resisting flavivirus invasion. TLRs are type I transmembrane proteins playing vital roles in recognizing pathogen-associated molecular patterns (PAMPs) achieved by binding leucine-rich repeats (LRR) [20]. In response to ligand binding, the intracellular toll-interleukin 1 (IL-1) receptor (TIR) domain activates intracellular singling through interaction with a family of adaptor proteins resulting in an inflammatory response and release of inflammatory cytokines [20, 21]. So far, twelve mice, ten humans [20], and ten avian functional TLRs [21] were identified; most TLRs are conserved at a degree of differential gene loss between diverse species. TLRs 1–10 are conserved between humans and mice; however, the precise function of TLR10 in mice has not been explored [22]. The avian TLRs differ significantly from mammals. Some avian TLRs could duplicate into two genes like TLR1La, TLR1Lb, TLR2a, and TLR2b [21]. From 10 avian TLRs, TLR2a, 2b, 3, 4, 5, and 7 are apparent orthologs to TLRs found in mammals, while avian TLR21 may be orthologous to TLR21 found in fish and amphibians, and TLRs 1LA, 1LB, and 15 are avian specific [21]. As another vital member of PRRs, RLRs also have essential roles in recognizing bacterial and virus as TLRs do. Two primary members of RLRs, RIG-I and MDA5 (melanoma differentiation-associated gene 5) [23], can identify RNA viruses in the cells and induce type I interferon and immune factors [24] while another member of RLRs, LGP2, can only regularize the signaling effect of MDA5 and RIG-I [25]. There are differences between species. MDA5 is widespread in mammals and avians. However, RIG-I is lost in chickens [26]. Moreover, because of the lack of RIG-I, chicken MDA5 will identify short dsRNA first, but duck and mammal MDA5 will recognize long dsRNA or synthetic dsRNA in advance, while RIG-I will locate short dsRNA like poly(I:C) [27].

**Figure 1:**
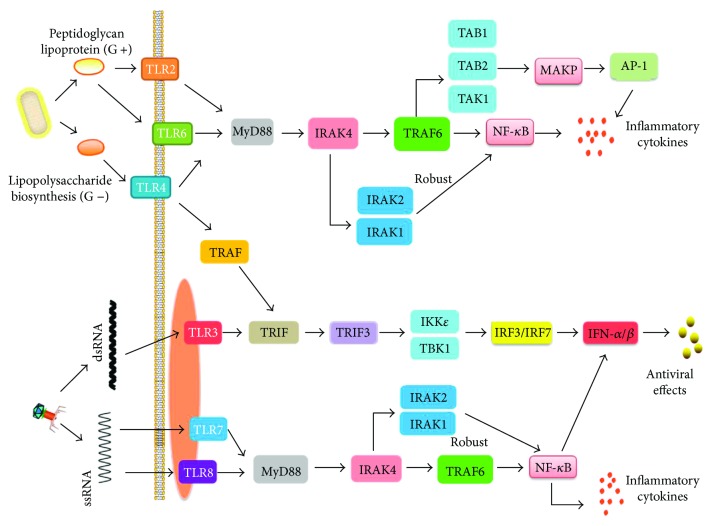
Toll-like receptor signaling pathway.

The TLR pathway is critical to combat and clearing pathogens, which contains two ways based on whether it has adaptor factor myeloid differentiation factor 88 (MyD88) or not. Based on reports, all TLRs, except TLR3, are targeting immune reaction through the MyD88-dependent pathway, while TLR3 targets the MyD88-independent pathway (TRIF pathway) to promote inflammatory cytokine product [20]. However, TLR4 is unique, which can target the TRIF pathway through interacting with recombinant translocation-associated membrane protein (TRAM) [28]. The MyD88-dependent pathway culminates in the activation of both NF-*κ*B and MAPK. After TLRs engage with PAMPs, the IL-1 receptor-associated kinases IRAK4, IRAK1, IRAK2, and IRAK-M are recruited by MyD88 [29]. IRAK4 is activated initially and is essential for activating NF-*κ*B and MAPK downstream of MyD88; consequently, IRAK1 and IRAK2 are activated which can cause robust activation of NF-*κ*B and MAPK when both of them are activated [29]. Another necessary factor to target NF-*κ*B is IKK complex, which can be polyubiquitinated by the TAK1 compound that can be activated through TRAF6 interacting with IRAK1, then enabled [30]. Simultaneously, TAK1 phosphorylates MAPK kinases, then various transcription factors are induced which include AP-1 [22]. The activation of the MyD88-dependent pathway results in producing various inflammatory factors and chemotactic effects. Through the TRIF-dependent pathway, both IRF3 and NF-*κ*B are activated [31], which ultimately induce various inflammatory factors and type I IFN. The mechanisms of TRIF-activated NF-*κ*B are similar to those of the MyD88-dependent pathway [22], while the mechanisms of IRF3 activation and interferon-*β* (IFN-*β*) transcription are particular. IRF3 can be phosphorylated and nuclear translocated by IKK, TBK1, and IKKi (IKK*ε*) that can be activated by TRIF requiring TRAF3 (Figure 1) [32, 33].

The RIG-I and MDA5 pathway will finally induce type I IFN. Commonly with RIG-I pathway, after MDA5 recognizes ssRNA or dsRNA virus, MDA5 can combine with the vital protein of mitochondrial MAVS (mitochondrial antiviral signaling protein) [34]. Then, MAVS is positioned on the mitochondrial outer membrane and recruits TRAF3, which can phosphorylate IRF3/IRF7 (IFN regulatory factor) when ubiquitin is terminated which starts the type I IFN antiviral immune response [34]. Moreover, this reaction will not happen when MAVS is positioned on the peroxisomes [35]. However, unlike human MDA5, the CARD domain of duck MDA5 can only target IRF7 while overexpression of CARD or the c-terminal RD-composed domain of MDA5 can induce the expression of IFN-*β* promoter [36].

Moreover, the pathway of TLR and RLR is complicated, which not only refers to TLRs and RLRs but also involves other molecules like microRNA (miRNA). There is abundant research that has reported that miRNA affects the process of TLR [37] and RLR [38] pathways during pathogen invasion through direct or indirect interaction with TLRs or RLRs. According to the reports, one miRNA can regulate more than one target gene [39]. On the contrary, one target gene can be regulated by numerous miRNAs [40, 41]; this is because miRNA does not need to combine the mRNA 3′ untranslated region (3′ UTR) of the target gene completely [42], which makes the regulating mechanism of TLR and RLR more complicated. Thus, figuring out the mechanism of how TLRs or RLRs combat pathogens is important.

## 2. The Function of TLRs in Resisting Flavivirus

### 2.1. TLR3 Plays Dual Functions during Flavivirus Infection

TLR3 has been found on the endosome of cells and shares the same function both in mammals and avians, which recognizes double-stranded RNA (dsRNA) and induces type I IFN [43, 44]. During flavivirus infection, TLR3 plays dual functions. On the one hand, TLR3 promotes host-immune process. Compared to other TLRs, TLR3 suppresses the replication of DENV more efficiently. After infecting DENV, the expression of human TLR3 (huTLR3) [45], IFN-*α*, IFN-*β*, and type III IFN (IL-28A/B) [46] significantly increased while the virus copy number decreased [47]. In further research, Tsai et al. and Chen et al. showed the high level of IFN-*α*/*β* produced from the TLR3-IRF3/IRF7 pathway [47, 48], and IFN-*β* is the reason for inhibiting DENV replication [46]. In HUH-7 cells, huTLR3 can recognize DENV-1 and induce the expression of IFN-*β*, which can enhance the expression of huTLR3 on the contrary [45]. TLR3 also induces type I IFN during WNV [49], ATMUV [50], and ZIKV [51] infections; however, the NS1 protein of WNV can suppress this way by inhibiting the transcription of IRF3 and activation of NF-*κ*B cytokine transcription [52], resulting in low levels of inflammatory factors. ATMUV is highly pathogenic to avians especially to ducks; during ATMUV infection of DEF cell and 293T cell, the expression of both duck TLR3 and huTLR3, respectively, and other inflammatory cytokines like IL-2, IL-6, and IL-29 are raised [50, 53]. Moreover, huTLR3 was found to suppress ATMUV replication through the TLR3/IRF3/IRF7 pathway in 293T cells [53]; however, the exact process of how TLR3 combats ZIKV has not been explored yet [51]. Also, the high level of huTLR3 detected in epidermis cutin cells and skin fibroblast cells following WNV infection indicates that huTLR3 may have an important function in skin immunity [54], but the specific mechanism has not been illuminated. On the other hand, TLR3 helps WNV invade the brain tissue and promotes WNV replication in the nervous system [55, 56]. Besides, infecting TLR3/7/8 mixed agonists or inhibiting the expression of anti-Fcc*γ*I or anti-Fcc*γ*IIa antibody will increase the expression of inflammatory cytokines while reducing the proliferation of DENV [57].

### 2.2. TLR7 Plays Immune Roles in Somatic Cells and Skin during Flavivirus Infection

The structure of TLR7 is similar in both mammals and avians; however, whether the function of TLR7 is likely between different species has not been clear [58]. In mammals, TLR7 expresses on endosome which can recognize single-stranded RNA (ssRNA). Moreover, in the immune process, TLR7 can not only recognize and combine with GU-rich single-stranded RNA and then induce type I IFN and cytokines [59] but it can also recognize synthetic poly(U) RNA and certain small interfering RNAs as well [60]. Albeit there is poor research studying avian TLR7, some reports found avian TLR7 have the function of combat virus [61]. During JEV and WNV infection, huTLR7 has been explored to play an important role. Mouse TLR7 is found to enhance the host immunity through interaction with JEV [62]. The expression of mouse TLR7 accompanied with CD80, CD86, and CD273 on the dendritic cell was significantly upregulated after JEV infection [62]. Intriguingly, suppressing TLR7 in mice increases the expression of TLR8, which indicates the high level of TLR8 making up for the loss of immunity that TLR7 targets [62]. Like the NS of DENV, some JEV's NS also assists JEV to escape the immune system. NS5 protein of JEV can inhibit the activation of IRF3 and NF-*κ*B, then reduce the amount of type I IFN and promote JEV replication process, which results from NS5 protein interaction with KPNA3 and KPNA4 [63]. TLR7 was also found to be necessary for skin immunity. WNV infecting Langerhans cells promotes IFN-*α*, IL-1*β*, IL-6, and IL-12 expression, but the same result was not detected after wiping off TLR7 [64]. Except to assume a role in skin immunity, TLR7 can target T cell answer as well. In Xie et al.'s experiment, wiping off MyD88 or TLR7 during WNV infection in mice could downregulate the expression of IgM and T cells, while the antigen presentation ability of DC was suppressed [65]. Intriguingly, infecting those mice again, the MyD88-lacking mice cannot target T cell answer, while TLR7-lacking mice appear to have a normal T cell answer, which suggests TLR7-MyD88 pathway is involved in resisting WNV process through activating T cell answer [65].

### 2.3. TLR2/TLR4/TLR6/TLR8 Resisting Flavivirus in Different Ways

TLR2, TLR4, and TLR6 can recognize both bacteria and virus, whereas TLR8 can only recognize single-stranded RNA (ssRNA) virus [66]. In avians, both TLR2 and TLR4 can recognize lipopolysaccharide (LPS) and then target NF-*κ*B [67, 68]. However, avians do not have TLR6 and TLR8; even though researchers found TLR8 in ducks, the structure of it is crushed which means it has no real function [69]. Albeit there are few pieces of research focusing on their roles in resisting flavivirus, they do serve important roles in the immune process. NS1 of DENV-1/3/4 can upregulate the expression of TLR2, TLR4, and TLR6 of humans [70, 71], while DENV-2 can only increase the huTLR2 expression [72]. The interaction of DENV-2 with a host will induce two consequences. For one thing, DENV-2 can target innate immunity through PI3K/NF-*κ*B pathway; for another, it can suppress the activation of NF-*κ*B as well, which reduces an amount of inflammatory cytokine secretion such as INF-*β*, IL-10, and IL-12 [73]. Cross-presentation system plays a vital role in recognizing the antigen and intercellular communication; however, JEV can inhibit this system to suppress TLR2-MyD88 and p38 MAPK pathway inducing CD8+ T cells [74]. Although TLR2 is required for combating JEV, the lack of TLR4 is beneficial for host immunity. Wiping off TLR4 enhances the expression of RIG-I, MDA5, PKR, Oas1, ISG49, ISG54, and ISG56 that can target IRF3 and NF-*κ*B pathway to induce inflammatory factors [75]. Otherwise, some other innate immune factors enhance the ability of DENV to infect the host. Cutting off the interaction between the antigen-antibody complex and FcR can reduce the expression of cytokines involved in TLR pathway and weaken the defensive ability of the host through upregulating SARM, TANK, and a negative regulation factor of the NF-*κ*B pathway, which results in a more efficient reproduction of DENV [57].

## 3. The Function of RLRs in Resisting Flavivirus

Except TLRs, RLRs are also detected to participate in resisting DENV processes, especially RIG-I and MDA5, which can identify RNA viruses in the cells and induce type I IFN and immune factors [24]. DENV infection can target a strong immune response through RIG-I/IPS-1/TBK1/IRF3 and MDA5 pathway [76], which can be strengthened by OAS2 [77]. OAS2 is an essential protein involved in the innate immune response to viral infection [78] and detected to be upregulated following DENV infection in keratinocytes, which cuts DENV RNA into more smaller debris that can be recognized by MDA5 and RIG-I and then enhances immune ability of the host [77]. However, the NS2A and NS4B of DENV-4 inhibit RIG-I/MDA5/TBK1/IKK*ε* immune pathway and phosphorylation of IRF3, in which process the NS4B-∆-118-260 of NS4B was identified to be indispensable [79]. NS2B/3 and NS4A of DENV help DENV escape RLR immunization. Among them, NS2B/3 can inhibit the phosphorylation of serine 386 and nuclear transfer of IRF3, which result from the interaction between NS2B/3 protein enzyme and IKK*ε* [80], while the NS4A of DENV-1 inhibits RIG-I and TBK1 inducing IFN-*β* [80]. Some immune processes also can promote DENV infection like antibody-dependent enhancement (ADE), which inhibits the activation of RLR-MAVS pathway by enhancing autophagy resulting in a more efficient replication of DENV [81]. RIG-I can recognize JEV in the different environment, like body cells and the nervous system [82, 83], which can secrete inflammatory factors like IL-6, IL-12p70, MCP-1, IP-10, and TNF-*α* to combat JEV infection [63]. RIG-I and MDA5 were found to be involved in resisting ZIKV and ATMUV infection; for the further study, Chen et al. found that type III IFN that was produced from the RIG-I/IRF3/IRF7 pathway can suppress ATMUV replication [53]. However, the specific process of how RIG-I and MDA5 combat ZIKV has not been explored yet [50, 51, 53].

## 4. Conclusion and Future Perspectives

Since TLRs and RLRs are the essential members of innate immunity and innate immunity is important in the early immune process, illuminating their function and mechanism on resisting flavivirus is necessary for further studies. However, for now, we only know which TLRs or RLRs take part in resisting flavivirus; the specific mechanism is not clear; therefore, there is more work for us to do in the future. Moreover, since the pathway of TLR and RLR is not only referring to TLRs and RLRs but also involving other molecules like miRNA, exploring and illuminating the function of miRNA or other molecules in innate immunity during pathogen invasion are also important in the future.
